# Polymeric nanomedicines for the treatment of hepatic diseases

**DOI:** 10.1186/s12951-022-01708-y

**Published:** 2022-11-19

**Authors:** Feixiang Luo, Ying Yu, Mingqian Li, Yuguo Chen, Peng Zhang, Chunsheng Xiao, Guoyue Lv

**Affiliations:** 1 Department of Hepatobiliary and Pancreatic Surgery, The First Hospital of Jilin University, Changchun, 130021 People’s Republic of China; 2 Key Laboratory of Polymer Ecomaterials, Changchun Institute of Applied Chemistry, Chinese Academy of Sciences, Changchun, 130022 People’s Republic of China

**Keywords:** Polymeric nanomedicines, Liver targeting, Hepatitis B virus, Nonalcoholic steatohepatitis, Hepatic fibrosis, Hepatocellular carcinoma, Host-versus-graft disease

## Abstract

The liver is an important organ in the human body and performs many functions, such as digestion, detoxification, metabolism, immune responses, and vitamin and mineral storage. Therefore, disorders of liver functions triggered by various hepatic diseases, including hepatitis B virus infection, nonalcoholic steatohepatitis, hepatic fibrosis, hepatocellular carcinoma, and transplant rejection, significantly threaten human health worldwide. Polymer-based nanomedicines, which can be easily engineered with ideal physicochemical characteristics and functions, have considerable merits, including contributions to improved therapeutic outcomes and reduced adverse effects of drugs, in the treatment of hepatic diseases compared to traditional therapeutic agents. This review describes liver anatomy and function, and liver targeting strategies, hepatic disease treatment applications and intrahepatic fates of polymeric nanomedicines. The challenges and outlooks of hepatic disease treatment with polymeric nanomedicines are also discussed.

## Introduction

The liver is the largest solid organ in the human body and performs a wide range of functions, including protein synthesis, metabolism, immune responses, endocrine-based regulation, biotransformation of nutrients, and detoxification. Therefore, disordered liver functions induced by various hepatic diseases, including viral hepatitis, fatty liver, hepatic fibrosis, hepatic cirrhosis, and hepatocellular carcinoma (HCC), significantly threaten human health, and result in nearly 2 million deaths per year around the world [1].

Various therapies have been applied in the clinical treatment of liver diseases, such as surgical resection, interventional therapy, chemotherapy, radiotherapy, antiviral therapy, immunotherapy, and liver transplantation (LT) [2, 3]. Although great achievements have be obtained in the clinic, some challenges limit the successful applications of current hepatic disease therapies. For example, incomplete resection may induce tumour recurrence; conventional pharmacotherapy may generate drug resistance, and result in severe systemic toxicity and limited therapeutic efficacy due to their lack of targeting ability; and the patients need to take immunosuppressant drugs for the remainder of their lives to avoid transplant rejection after LT, which may induce severe side effects and reduce their living quality [4, 5]. Especially, owing to the various and complex etiologies of nonalcoholic steatohepatitis and hepatic fibrosis, no standard medication is available for their clinical treatment despite the proved effectiveness of some therapeutic agents in clinical trials [6, 7]. Recently, nanomedicines have been reported to have the capacity to improve the therapeutic outcomes and reduce the side effects of medications (e.g., natural extracts, chemotherapeutic drugs and nucleic acid-based drugs) by facilitating liver-specific drug delivery, thus offering new paradigm to address the aforementioned challenges.

**Fig. 1:**
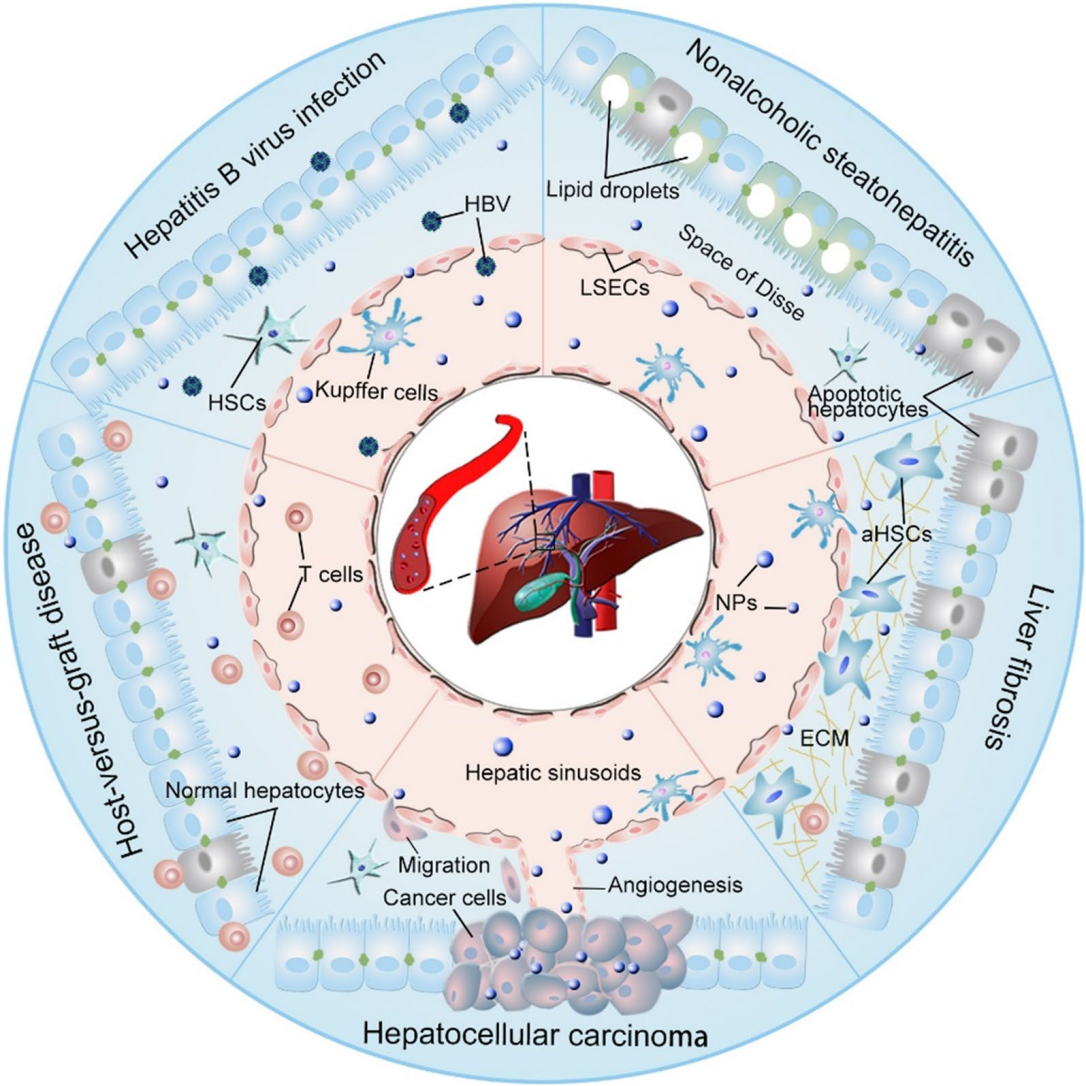
Scheme illustrating the application of polymeric nanomedicines in the treatment of various hepatic diseases, including hepatitis B virus infection, nonalcoholic steatohepatitis, hepatic fibrosis, hepatocellular carcinoma, and host-versus-graft disease

Significant progress has been made in the field of nanomedicine in terms of disease diagnosis and treatment in the past half century [8, 9]. Since Gregoriadis and coworkers developed the first liposomes in 1974 [10], various nanodrug delivery systems have been developed. These carriers typically include biomacromolecules (e.g., proteins), inorganic materials, viral capsids, polymers, and lipids. The excellent physicochemical properties of nanoparticles (NPs) provide them with superior advantages, including improved drug pharmacokinetics, cell and tissue gap penetration ability, enhanced drug accumulation at diseased sites by passive or active targeting strategies, controlled drug release, reversing multidrug resistance, enabling high contrast imaging, and reductions in side effects [11, 12]. The commercialization of nanodrugs (e.g., Caelyx/Doxil, and Smarticles) has led to great successes in tumour therapy and in infectious disease therapy (e.g., Ambisome^®^) [12, 13]. Furthermore, nanomedicines based on various materials have been developed to treat hepatic diseases [12, 14]. Among them, lipid- and polymer-based nanomedicines have been used most frequently due to their well-established synthesis and characterization methods, high biocompatibility, and biodegradability [11]. However, the further development of lipid nanomedicines is restricted to some extent by their limited stability (e.g., the fragile nature of lipid bilayer of liposomes) and difficulties in functionalization of lipids [15]. Polymeric nanomedicines, in contrast, can be easily modified to attain ideal physicochemical characteristics and functions, such as high stability, stimuli-responsive properties, and targeting ability, and therefore have been widely developed for the treatment of various hepatic diseases, such as hepatitis B virus (HBV) infection, nonalcoholic steatohepatitis (NASH), liver fibrosis, HCC, and host-versus-graft disease (HVGD) (Fig. 1) [11].

**Fig. 2:**
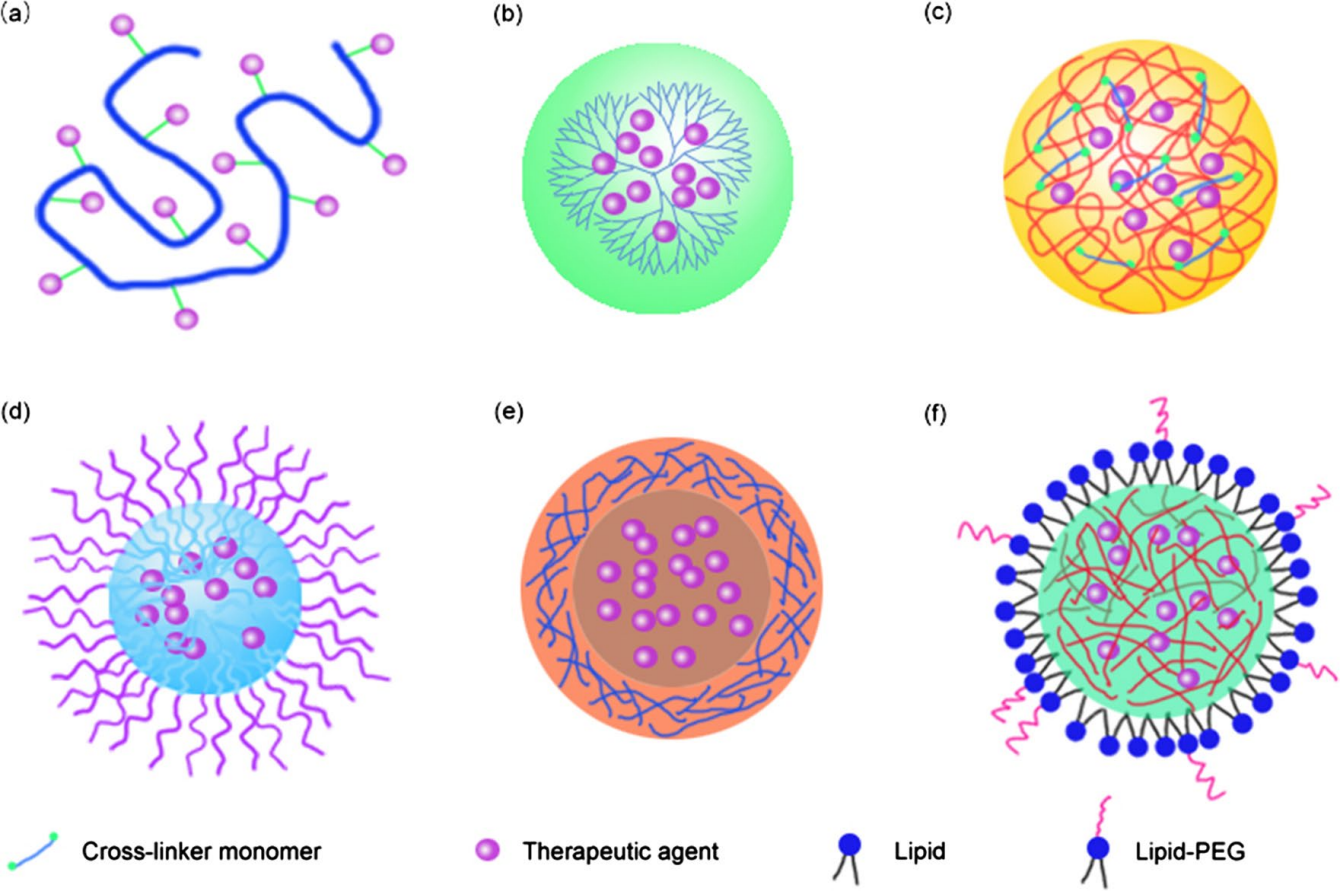
Structural illustration of polymer-based nanoplatforms. **a** Polymer conjugates, **b** dendrimers, **c** nanogels, **d** micelles, **e** nanocapsules, and **f** lipid-polymer hybrid nanoparticles

Polymer-based nanomedicines can be roughly classified into several categories: polymer conjugates, dendrimers, nanogels, polymeric micelles, polymeric nanocapsules, and lipid-polymer hybrid NPs (Fig. 2) [16]. Some polymer NPs have been approved for patient use. For example, Zoladex, a poly(lactic-*co*-glycolic acid) (PLGA) copolymer carrying goserelin acetate for the treatment of breast cancer and prostate cancer, was approved in 1998. Trelstar Depot, a triptorelin pamoate microparticle with a PLGA carrier for use in the treatment of advanced prostate cancer, was approved in 2000. A paclitaxel polymeric micelle (Genexol-PM) used for treating malignant tumours was approved in Korea in 2007 [17, 18]. Although several polymeric nanomedicines for the treatment of HCC and NAFLD underwent clinical trials, none of them have yet been approved (Table 1). Therefore, great efforts continue to be needed to promote the development of polymer-based nanomedicines for the treatment of hepatic diseases. This review describes liver anatomy and function, and liver targeting strategies, hepatic disease treatment applications, and the intrahepatic fates of polymer-based nanomedicines. The challenges and outlooks for effective therapy of hepatic diseases using polymeric nanomedicines are also discussed.

**Table 1:** List of clinical trials of polymeric nanomedicines for the treatment of liver diseases (clinicaltrials.gov)

Cause	Name	Payload name	Carrier	Year of starting/completion	Study phase	No. of patients	Status	Clinical trials identifier
HCC	Genexol-PM	Paclitaxel	Polymeric micelle	2016/2021	2	5	Terminated	NCT03008512
HCC	Doxorubicin	Doxorubicin	Polymer microspheres	2010/2012	1/2	24	Completed	NCT01116635
HCC	Doxorubicin	Doxorubicin	Polymer microsphere	2016/2019	–	100	Recruiting	NCT02743065
HCC	PK2	Doxorubicin	HPMA drug conjugate	–	2	–	Completed	[19]
HCC	DHAD-PBCA-NPs	Mitoxantrone	Polybutylcyanacrylate nanoparticles	–/2009	2	108	Completed	[20]
HCC	Lipotecan	TLC388 (Camptothecin derivate)	Polymeric micelle	2014/2015	2	29	Terminated	NCT02267213
HCC	Doxorubicin Transdrug	Doxorubicin	Poly(isohexyl cyanoacrylate) nanoparticle	2012/2019	3	397	Completed	NCT01655693
NAFLD	Nanocurcumin	Curcumin	Polymeric nanoparticle	2016/2017	–	84	Completed	[21]

HCC, hepatocellular carcinoma; HPMA, N-(2-hydroxypropyl) methacrylamide; DHAD, dihydroxyanthracenedione; PBCA, polybutylcyanacrylate; NPs, nanoparticles; NAFLD, nonalcoholic fatty liver disease

## Hepatic anatomy and function

**Fig. 3:**
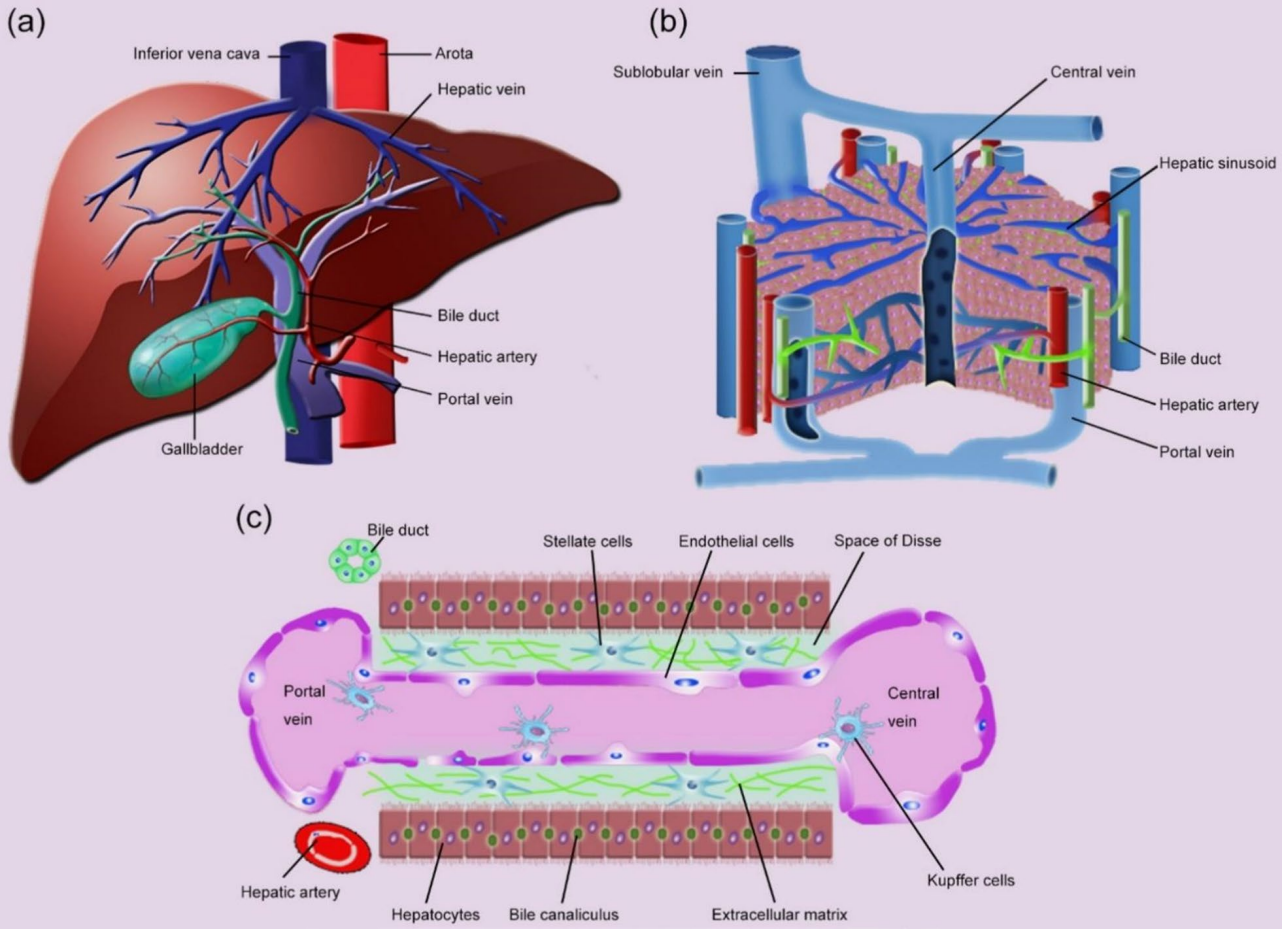
Structure of the liver. **a** The blood and bile systems of the liver. **b** The hepatic lobule is a functional and structural unit of the liver. **c** The hepatic sinusoid is the main area of material exchange in the liver

**Fig. 4:**
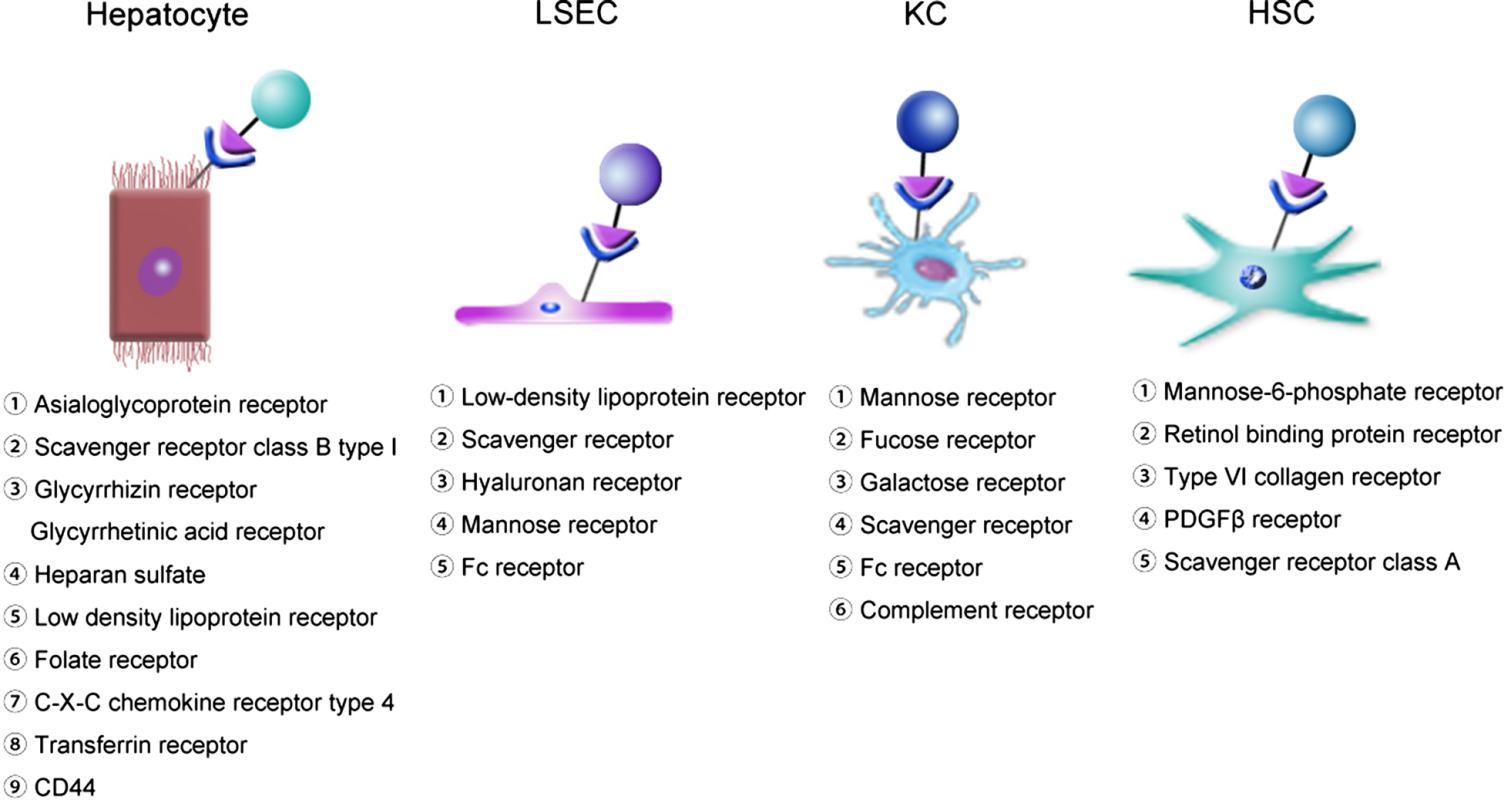
List of specific receptors expressed on different liver cell types

The liver is under the ribcage on the right-hand side of the abdomen (Fig. 3a), and accounts for approximately 2–3% of the body weight [22]. The blood of the liver is supplied mainly by the hepatic artery which contributes 25% of the liver blood supply but 75% of the oxygen, and by the portal vein which contributes 75% of the supplied blood (Fig. 3a). Typically, the liver consists of parenchymal cells and nonparenchymal  cell (NPCs). The parenchymal cells include hepatocytes, which account for 70–80% of the cells in the liver and are the principal cell type in liver [23]. The NPCs mainly include liver sinusoidal endothelial cells (LSECs), Kupffer cells (KCs), and hepatic stellate cells (HSCs) [24]. LSECs form the lining of the hepatic sinusoid and have fenestra with pore size of approximately 100–200 nm [25, 26]. KCs account for 80–90% of the total number of macrophages in the body, and are intrinsic parts of the reticuloendothelial system (RES). HSCs make up nearly one-third of the NPCs [27, 28], reside in the space of Disse, and directly contact LESCs, hepatocytes, and other HSCs [29]. The detailed functions of parenchymal cells and NPCs are shown in Table 2, together with various hepatic diseases associated with different cell types. Furthermore, the diseased cells overexpress some specific receptors that can be used for targeted therapy of according hepatic diseases (Fig. 4).

**Table 2:** Characteristics of liver cells and the common diseases associated with each cell type

Live cell type		Functions	Related diseases
Parenchymal cells	Hepatocytes	Lipid, protein, and carbohydrate metabolism Detoxification Immune/inflammatory response Secretion of lipoproteins, coagulation factor, growth factors, and cytokines Ammonia and urea biosynthesis Synthesis of cholesterol, bile salts and phospholipids Start of bile formation and secretion	Viral hepatitis Alcohol-induced steatohepatitis (ASH) Non-alcohol steatohepatitis HCC Autoimmune diseases Wilson’s disease Hemochromatosis α1 antitrypsin deficiency
Non-parenchymal cells	LESCs	Physical barrier and host defense Scavenger function Immune/inflammatory responses Secretion of cytokines Remove foreign materials and macromolecular waste	Hepatic veno-occlusive disease
	KCs	Host defense Immune/inflammatory response Detoxification Secretion of cytokines, chemokines, growth factors, and proteolytic enzymes Maintaining functional iron metabolism and bilirubin metabolism Control cholesterol metabolism	HBV or HCV Acute liver failure Chronic liver injury Metabolic and alcoholic liver disease NASH HCC Cholestatic liver diseases Liver fibrosis
	HSCs	Vitamin A and lipid metabolism Detoxification Immune/inflammatory responses Secretion of lipoproteins, growth factors, and cytokines	Liver fibrosis and cirrhosis ASH, NASH, HCC

HCC, hepatocellular carcinoma; LESCs, liver sinusoidal endothelial cells; KCs, Kupffer cells; HBV, hepatitis B virus; HCV, hepatitis C virus; NASH, nonalcoholic steatohepatitis; HSCs, hepatic stellate cells; ASH, alcohol-induced steatohepatitis

The functional and structural unit of the liver is a hepatic lobule, which measures approximately 2.0 × 0.7 mm and is composed of hepatocyte plates organized in a solid hexagonal shape surrounding the central vein. It is separated by the anastomosing system of sinusoids that perfuse cells with a mixture of portal and arterial blood. A portal triad containing the hepatic artery, the portal vein, and the bile ducts is arranged around each vertex of the hepatocyte-formed hexagon (Fig. 3b) [30–32]. Moreover, the hepatic sinusoid has a diameter of approximately 5 to 10 μm, and could facilitate the exchange of substances between the blood and the perisinusoidal space of Disse (Fig. 3c) [30].

## Strategies for hepatic-targeted drug delivery

**Fig. 5:**
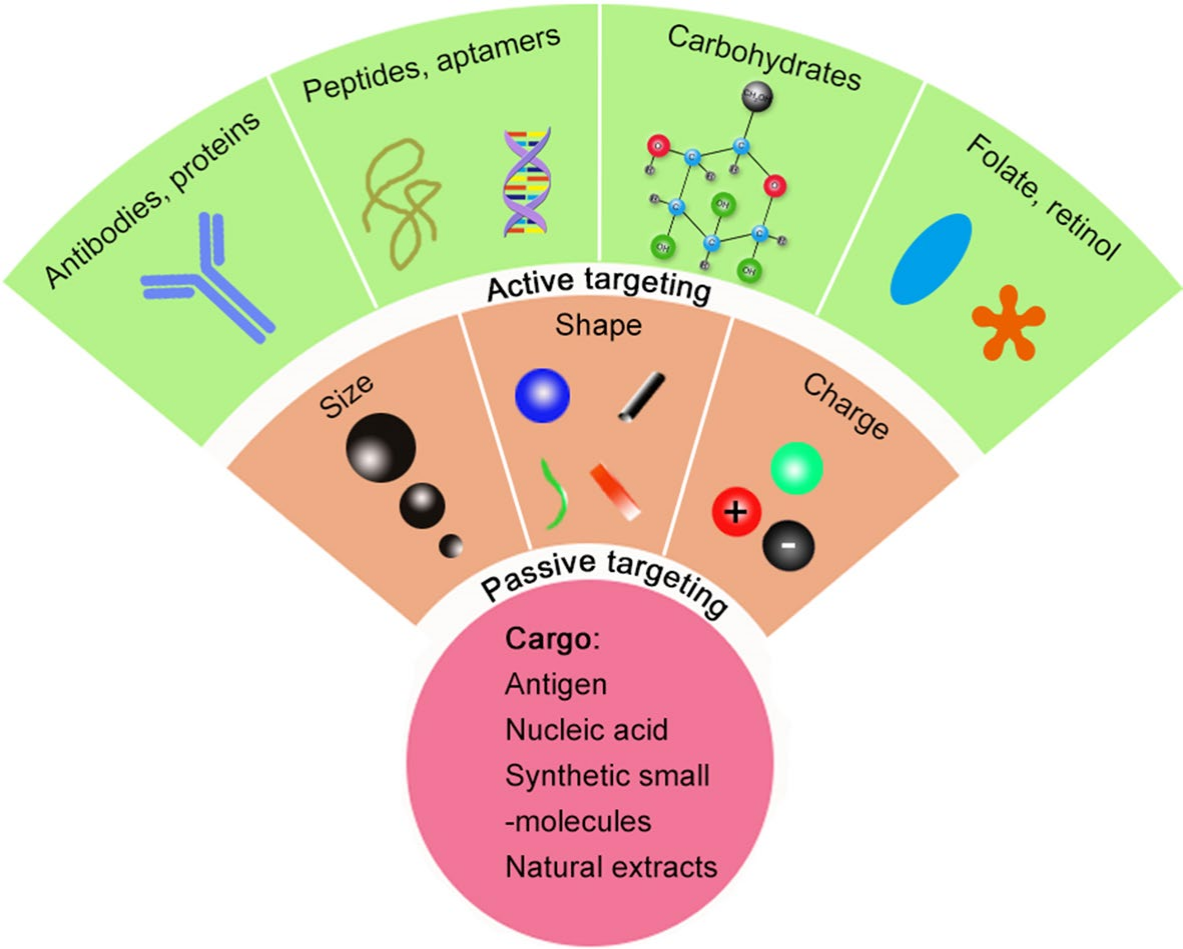
Strategies for targeting nanomedicines loaded with various cargoes to the liver. Nanomedicines first enter the liver by passive targeting, and then are internalized by liver cells through ligand-mediated endocytosis (active targeting)

To treat hepatic diseases precisely, liver-targeted drug delivery systems have been developed and have made considerable progress in this area. In summary, nanomedicines can passively or actively target disease sites. The passive accumulation of NPs in the liver depends on the properties of the NPs, structure of hepatic lobules, pathophysiological features of liver diseases, and mode of drug administration. Passive accumulation of nanomedicine in target tissues increases local drug concentration and uptake by diseased cells, thereby reducing drug distribution to healthy organs. Active targeting, however, depends on the cell-specific ligands decorating the surface of NPs, because these ligands can recognize and bind to the specific receptors on certain types of cells, thereby preventing unspecific toxicity on normal liver cells. In fact, these two targeting strategies are often performed concurrently; NPs first enter the liver by passive diffusion and are subsequently internalized through receptor-mediated endocytosis (Fig. 5).

### Passive targeting

Following intravenous administration, NPs that carry a significant surface charge may be rapidly covered with a layer of proteins and may elicit immune responses, leading to their uptake by KCs in sinusoids [33, 34]. Research has shown that NPs with negatively charged surfaces are taken up at an increased rate by the RES in the liver, while hepatocytes are prone to internalize NPs with cationic surface charges [12, 34]. In addition, compared to hydrophilic NPs, hydrophobic NPs are more rapidly eliminated from blood by the RES [34]. Therefore, NPs are often polyethylene glycol (PEG)ylated to prolong their blood circulation, reduce protein binding, and minimize their uptake by the RES.

In addition, NP size plays a crucial role in the targeted therapy of hepatic diseases. NPs with a diameter smaller than 200 nm and without being taken up by KCs can enter the space of Disse through slightly larger-than-average fenestrations, through which they diffuse to various liver cells [11]. The smaller the size of the NPs is, the easier they pass through the fenestrations deep into the space of Disse and are internalized by hepatocytes [35]. Surprisingly, studies have shown that nanocarriers as large as 400 nm can enter the space of Disse and pass through the fenestrations via a mechanism of forced extrusion [36]. The shape of designed NPs can also affect NP distribution. Rod-shaped and worm-like NPs with very high aspect ratios are not taken up as rapidly by KCs as spherical shaped NPs [37, 38].

Passive accumulation of nanomedicine in some solid tumours can be realized through the enhanced permeability and retention (EPR) effect, which is caused mainly by the immature differentiation of tumour vasculature and impaired lymphatic drainage [39–42]. The diameter of the fenestrations is reported to vary between 400 and 600 nm in human tumours [43]. Therefore, NPs are also widely used in passive targeted therapy of HCC by EPR effect [44–46]. In addition, some studies have indicated that the optimal diameter of NPs used for cancer treatment is between 70 and 200 nm [47].

In addition to particle properties, the structure of hepatic lobules, the pathophysiological features of HCC, and the method and site of administration also affect liver cell uptake and intrahepatic distribution of nanomedicines. For example, some methods, including hydrodynamic injections, and intra-arterial, intratumoral and intrabiliary infusions, have been employed to facilitate the accurate diagnosis and treatment of liver diseases [11, 12].

### Active targeting

Active targeting, also known as ligand–receptor-mediated targeting, involves the use of peripherally conjugated targeting moieties for the specific uptake of nanomedicines by target cells [42, 48]. Targeting moieties, such as antibodies, proteins, peptides, aptamers, carbohydrates, and vitamins (e.g., folate and retinol) (Table 3), can target surface molecules or receptors overexpressed on disease cells [48, 49]. As mentioned above, various receptors are expressed on the surface of liver cells and can be used to develop liver-targeted nanomedicines (Table 3).

**Table 3:** Reported cellular receptors and corresponding ligands in liver-targeted drug delivery

Liver cell type	Cellular target	Targeting ligand	References
Hepatocytes	Asialoglycoprotein receptor	Galactose	[50, 51]
		*N*-acetyl galactosamine	[52]
		Asialofetuin	[53]
		PreS1 peptide	[54]
		Lactobionic acid	[55, 56]
		Lactosyl-norcantharidin	[57]
		Soybean sterylglucoside	[58]
		Pullulan	[27]
	Scavenger receptor class B type I	Apo A-I	[28]
	Glycyrrhizin receptors Glycyrrhetinic acid receptor	Glycyrrhizin Glycyrrhetinic acid	[29] [55, 59, 60]
	Heparan sulfate	CKNEKKNKIERNNKLKQPP peptide	[61]
	Low density lipoprotein receptor	Apo E and ApoB100	[62, 63]
	Folate receptor	Folate	[51, 64, 65]
	C-X-C chemokine receptor type 4	Plerixafor	[66]
	Transferrin receptors	DT7 and LT7 peptides, transferrin	[67]
	CD44	Hyaluronan	[68]
LESCs	Low-density lipoprotein receptor	KLGR peptide	[69]
	Scavenger receptor	Aconitylated human serum albumin	[70]
		LPS and oxidized or acetylated LDL	[71]
	Hyaluronan receptor	Hyarluronic acid stearylamine	[72]
	Mannose receptors	Mannose/*N*-acetylglucosamine	[73, 74]
		Gelatin and collagen a-chain	[75]
	Fc receptors	IgG and IgE	[76]
KCs	Mannose receptors	Mannose	[77]
		4-Aminophenyl-α-d-mannopyranoside	[78]
	Fucose receptors	Fucose	[79]
	Galactose receptors	Galactose	[80]
	Scavenger receptors	IgG and calcitriol	[81]
		LPS and oxidized or acetylated LDL	[71]
	Fc receptors	CC52 antibody	[82]
	Complement receptors	Complement fragments (e.g. C3b and iC3b)	[83]
HSCs	Mannose-6-phosphate receptor	Mannose 6-phosphate	[84]
	Retinol binding protein receptor	Retinol	[85] [86, 87]
	Type VI collagen receptor	cRGD* peptide	[88, 89]
	PDGFβ receptor	pPB	[90, 91]
	Scavenger receptor class A	M6P-HSA	[92]

Apo, apolipoprotein; LPS, lipopolysaccharide; LDL, low density lipoprotein; cRGD, cyclic Arginine-Glycine-aspartic acid; PDGFβ, platelet derived growth factor β; pPB, cyclic PDGFβR-recognizing peptides; M6P-HAS, mannose 6-phosphate human serum albumin

The concept of targeted NPs has been implemented for more than 40 years, but only a minority of these NPs have entered into clinical trials, and none are currently approved for clinical application [17]. Many factors influence the targeting ability of NPs, including (i) the administration route of the NPs, such as the oral, intramuscular, intravenous, or intratumoral routes, and (ii) the physicochemical properties of the NPs, such as the types and density of the ligands, as well as the size, materials, shape, charge, and surface hydrophobicity of the NPs [42]. Therefore, improvement of the active targeting ability of nanomedicines is needed to promote their clinical translation.

## Polymer-based nanomedicines for liver disease treatment

Liver diseases are related to pathological changes in the structure and function of liver cells, which are usually induced by endogenous or exogenous pathogenic factors [93–96]. Common liver diseases associated with different cell types are summarized in Table 2. Based on the current understanding of pathophysiological mechanisms of hepatic diseases at the cellular level and the understanding of the macroscopic and microscopic anatomy of the liver, researchers have designed many drug delivery systems in recent years, and they have shown excellent application possibilities. In the following section, we review the progress in the development of various polymer-based nanocarriers that show promises for the specific delivery of therapeutic agents to treat HBV infection, NASH, hepatic fibrosis, HCC, and HVGD after LT (Table 4).

**Table 4:** Polymer-based NPs as drug carriers for liver disease treatment

Liver disease	Carrier	Delivered drug	Size (nm)	References
HBV infection		HBsAg	143 ± 33	[97]
		siRNA	60	[98]
		Bay41-4109	300–400	[99]
		HBsAg	186.6 ± 3.7	[77]
		DrzBC and DrzBS	123.00 ± 10.98	[100]
		HBsAg	206.3 ± 4.20 and 253.1 ± 7.75	[101]
NAFLD			132.6 ± 13.5	[102]
		IL-22	100	[103]
			50	[104]
	PLGA		210	[105]
	PLGA	Resveratrol	176.1	[106]
	PLGA		160	[107]
		miR-146b mimic	150–350	[108]
			57.7 ± 14.1	[109]
Liver fibrosis			/	[110]
	PLGA		187.6 ± 5.0	[111]
	CS		99 ± 22	[112]
	CS	Collagenase	90 ± 3	[113]
			120	[114]
			70–80	[84]
			632.28 ± 12.15	[115]
			300	[116]
	CS		200–250	[117]
		siRNA	40	[118]
HCC			144.7 ± 6.53	[119]
		Sorafenib	118.3	[120]
			127.96 ± 4.6	[59]
			188.4 ± 6.3	[29]
		Sorafenib	127	[121]
		Sorafenib	278.7 ± 2.2	[122]
			/	[123]
			242 ± 4.6	[124]
	HA	DOX	217.70 ± 0.89	[60]
		DOX	54.27–63.11	[125]
	PLGA		211	[126]
HVGD		shRNA	200–300	[127]
			110.9	[128]
		Tacrolimus	2.30 ± 0.14 μm	[129]
	CS, PLGA	Tacrolimus	345	[130]

HBV, hepatitis B virus; CS, chitosan; HBsAg, hepatitis B surface antigen; PLGA, poly(lactic-*co*-glycolic acid); mPEG, methoxypolyethylene glycols; NAFLD, nonalcoholic fatty liver disease; Lac-PDMAEMA, lactosylated poly(2-(dimethylamine)ethyl methacrylate)nanoparticle; PAMAM, polyamidoamine; PEG-PCD, poly (ethylene glycol)-block-poly (2-methyl-2-carboxyl-propylene carbonate)-graft-dodecanol; MEO_3_MA, tri(ethylene glycol) methylether methacrylate; PFPMA, pentafluorophenyl methacrylate; PAM-PBLG-*b*-TPGS, poly(amidoamine)-poly(γ-benzyl-l-Glutamate)-b-d-α-tocopheryl polyethylene glycol 1000 succinat; HZ, hydrazide; PLA, polylactic acid; GA, gycyrrhetinic acid; DOX, doxorubicin; PLys, poly(lysine); HA, hyaluronic acid; PBA, phenylboronic acid; Glu, glutamic acid; DA, dopamine; HGPAE, histidine-grafted poly(β-amino ester)

### Hepatitis

Hepatitis is characterized by structural and functional impairment of liver cells and the infiltration of inflammatory cells and inflammatory factors. Long-term alcoholism, exposure to toxic agents, and viral/bacterial infection can eventually lead to liver damage. HBV infection and NASH are the two most common chronic hepatic diseases, and no efficient therapy is available for them thus far. In recent years, many attempts have been made to improve the treatment effect of hepatitis.

#### HBV infection

HBV is a hepatotropic DNA virus that can cause a lifelong chronic infection. HBV replication in hepatocytes can cause liver fibrosis, cirrhosis, and HCC. Although HBV surface-antigen vaccines are effective in preventing HBV infection, it is difficult to eliminate HBV from infected cells. Various antiviral agents, including interferons, nucleic acids, and small molecules, have been used to control HBV infections. However, these therapeutics lead to dose-limiting side effects and may induce the development of drug resistance [131]. Various polymeric nanocarriers have been developed to deliver classical antiviral drugs (vaccines, nucleic acids, and small molecules) to hepatocytes for HBV infection treatment, which effectively reduce the side effects and enhance the therapeutic outcomes of drugs [8, 11].

(i) Vaccine delivery: The HBV vaccine refers to inactivated HBsAg, which is the mainstay of hepatitis B prevention. However, the poor absorbability, low immunogenicity, and poor patient compliance in receiving the traditional inoculation inhibit the further development of vaccines. Hence, some alternative routes of administration have been developed to eliminate these difficulties. The nasal mucosa contains a rich capillary plexus, through which the vaccines can enter the blood quickly; therefore, Subbiah et al. formulated a *N*,*N*,*N*-trimethyl chitosan (CS) nanocarrier to deliver HBsAg (N-TMC NPs) through the nasal route. The NPs increased the drug-loading rate and prolonged antigen release in vitro, and an immunological study revealed that the adjuvant efficiency of these NPs for the antigen was highly stable and better than the standard efficiency in vivo [97]. Similarly, nasal vaccination has also been performed with nanovaccines consisting of poly-ε-caprolactone (PCL)/CS and HBsAg based on the stability of PCL in the blood circulation and the mucoadhesive and immunostimulatory properties of CS. The combined delivery of PCL and CS produced a synergistic effect in the efficient generation of an immune response [132]. Furthermore, Dewangan et al. designed a variety of NPs based on PLGA to deliver HBsAg and utilized a central composite design for formulation optimization. The results showed that the intramuscular delivery of the nanovaccine resulted in stronger humoral and cellular responses [133, 134]. Zhu et al. loaded HBsAg onto mannose-modified PLGA NPs, which slowly released HBsAg and enhanced antigen presentation to lymphocytes, resulting in long-term immunity. The study showed that NP uptake by bone marrow-derived dendritic cells (BMDCs) and RAW 264.7 cells was significantly increased. In addition, subcutaneous delivery of NPs maintained humoral immunity and enhanced cellular immune responses in animal tests [77]. Moreover, Wang et al. prepared a HBsAg nanogel (Ng) using CS and poly-γ-glutamic acid (γ-PGA). The results indicated that HBsAg Ng not only boosted the immune system but also promoted the proliferation of memory T cells. Moreover, the positively charged Ng (+) was more stable and provided longer protection against HBV than the negatively charged Ng (−), making it desirable as a HBsAg vaccine carrier [101].
(ii) Nucleic acid delivery: In recent years, gene therapy has been widely applied to the treatment of HBV infections. CS and its derivatives have been widely used to fabricate gene delivery vectors [135]. For example, Zeng et al. fabricated NPs using only PLGA, and the NPs initially showed low capacity for plasmid DNA (pDNA) encapsulation; however, by combining PLGA with CS as a carrier, the retention of anionic pDNA, encapsulation efficiency and drug loading capacity were all increased. CS-modified PLGA NPs showed a positive zeta potential and were effectively taken up by the cells, which was conducive to antiretroviral therapy in vivo [98]. In addition, cell-penetrating peptides (CPPs) possess a significant capacity for membrane transduction and can deliver various bioactive molecules into cells. Therefore, CPPs combined with CS may be used as an ideal nonviral vector to deliver peptide nucleic acids (PNAs) or DNA vaccines to treat HBV [136]. DrzBC and DrzBS (10–23 DNAzymes) effectively inhibited the expression of HBV e- and s-genes, respectively, and greatly reduced viral load. Therefore, Miao et al. designed a CS-based glycolipid-like nanocarrier (CS oligosaccharide-SS-octadecylamine, CSSO) with redox-responsive and endosomal escape properties. This carrier bound with DrzBC and DrzBS DNA via electrostatic interactions to form CSSO/DrzBC and CSSO/DrzBS complexes. Studies showed that CSSO/DNA powerfully inhibited HBV replication [100].
(iii) Synthetic small-molecule delivery: Bay41-4109 is an effective inhibitor of HBV replication. Jiang and coworkers prepared Bay41-4109-loaded CS NPs. The NPs significantly improved the bioavailability of Bay41-4109 and provided an effective method for the treatment of HBV [99]. In addition, PLGA microspheres with the capacity of sustained release were prepared and loaded with adefovir and entecavir. The microspheres reduced the medication dose and frequency in patients with chronic hepatitis B [137–139]. Recently, Hamdi et al. synthesized lipid polymer hybrid (LPH) NPs with the merits of polymers and liposomes to deliver entecavir. The physicochemical properties of the vitamin E-modified LPH NPs were favourable and confirmed by related tests. The NPs targeted macrophages and showed enhanced the retention in J774 macrophage cells, thereby reducing viral replication in these macrophages [140].

#### Nonalcoholic steatohepatitis

NASH is an inflammatory subtype of nonalcoholic fatty liver disease (NAFLD), to which alcohol consumption is not a contributor [141–143]. The development of NASH involves many molecular pathways, and various pathogenic factors lead to highly heterogeneous diseases and clinical manifestations [144]. A widely accepted explanation involves the inability of the liver to catabolize an overabundance of carbohydrates and fatty acids, which leads to an increase of toxic lipid species in hepatocytes [6, 142, 145–147]. These primary metabolic energy substrates induce hepatocellular stress, injury, and death, leading to fibrogenesis and genomic instability, and increasing the risk of cirrhosis and HCC [142].

In recent years, with increasing awareness of the pathogenesis of NAFLD, great progress has been made in the research and development of various drugs, but significant challenges remain unresolved, and no drugs have been approved for clinical use. Fortunately, several medications have been proved to be effective at reducing steatohepatitis in clinical trials, including weight loss medications, insulin sensitizers, vitamin E, cholesterol-reducing medications, cytoprotective agents, and obeticholic acid [143]. However, the efficacy and safety of these drugs require further evaluation. To improve the drug treatment effect on NAFLD, novel polymeric nanomedicines have been recently developed.

**Fig. 6:**
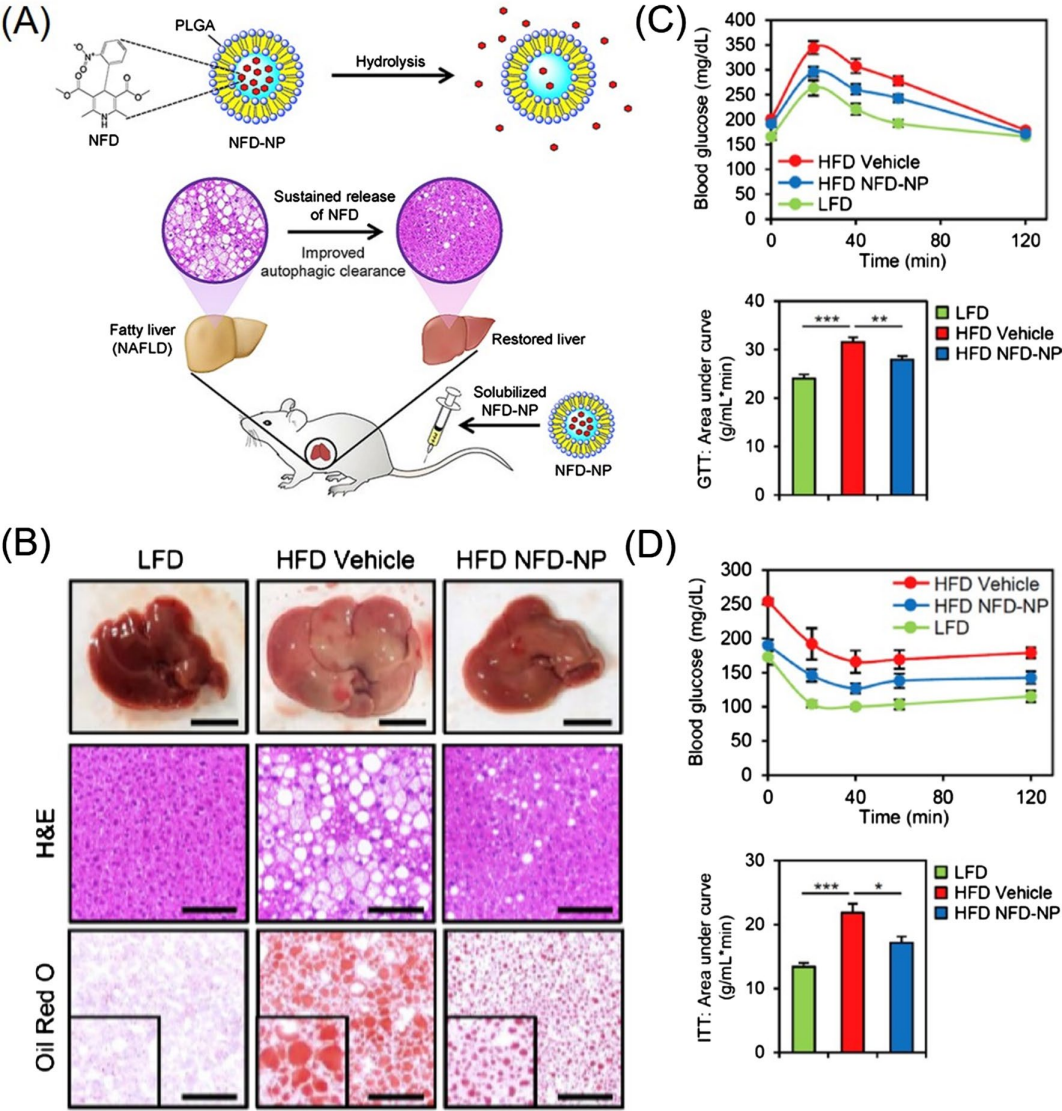
**A** Schematic illustration of Nifedipine nanoparticles (NFD-NPs) for preventing NAFLD. **B** NFD-NPs alleviate high-fat diet (HFD)-induced obesity and hepatic steatosis. **C** and **D** NFD-NPs improve insulin sensitivity and glucose tolerance. (Reprinted with permission from Ref. [105], Copyright 2019, Elsevier Ltd)
(iii) Nucleic acid delivery: IL-22, an effective agent for alleviating NAFLD, might induce severe adverse effects at high doses. Therefore, biguanide and CS were used to synthesize a novel polymetformin carrier that can be combined with penetratin and DSPE-PEG2000 to form stable nanocomplexes with the IL-22 gene. NPs containing IL-22 and metformin exhibited dual therapeutic effects on NAFLD, remarkably alleviating hepatic steatosis, restoring insulin sensitivity, and attenuating metabolic syndrome in animal tests [103]. Furthermore, a miR-146b mimic exhibited potent anti-inflammatory activity in NASH; therefore, the miR-146b mimic was targeted to hepatocytes in NAFLD mice with lactosylated poly(*N*,*N*-dimethylaminoethyl methacrylate) (Lac-PDMAEMA) as the carrier. These NPs effectively suppressed the expression of PPARγ and decreased the levels of TNF-α and IL-6 mRNA, ultimately alleviating hepatic steatosis [108].

(i) Natural extract delivery: Resveratrol (Res), a phytoalexin extracted from grapes and other food product, can regulate blood lipid and blood glucose homeostasis and relieve metabolic disorders. However, the application of Res is limited due to its poor bioavailability and stability. Therefore, researchers improved its physicochemical characteristics by loading it into a galactose (Gal)-modified polymer carrier. Results showed that the Res-loaded nanomedicines effectively prevented NAFLD progression compared with free Res [104, 106]. Both CS, a natural cationic aminopolysaccharide, and silymarin, a main component in milk thistle extracts, have the ability to lower lipids. Therefore, Liang et al. synthesized CS-modified, silymarin-loaded LPH NPs with a shell-core structure consisting of a polymer core and a phospholipid shell to enhance the oral bioavailability and improve the lipid-lowering efficacy of silymarin in NAFLD treatment [148].
(ii) Synthetic small-molecule delivery: De novo lipogenesis (DNL) is relevant to sterol regulatory element-binding protein (SREBP)-1c and is significantly increased in NAFLD patients. Therefore, DNL may be a potential therapeutic target. Zhao et al. prepared mPEG-PLGA loaded with rapamycin (RAPA) to treat NAFLD, which significantly decreased the lipid content, attenuated hepatic steatosis, and repaired liver injury in mice [102]. Cao et al. studied the therapeutic efficacy of biodegradable polyurethane NPs loaded with a PPARα agonist fenofibrate (FNB) on NAFLD. They demonstrated that the NPs dramatically reduced triglyceride levels both in vitro and in vivo, and increased the plasma FNB concentration of mice [109]. Nifedipine (NFD) suppressed the high-fat diet-induced accumulation of p62 and ubiquitinated protein inclusions by restoring cytosolic calcium homeostasis and inducing autophagy and lysosomal degradation. Recently, NFD-NPs that alleviated obesity-related metabolic dysfunction were developed using PLGA as the carrier. NFD-NPs increased the concentration of NFD and restored lipid metabolism associated with NAFLD by enhancing autophagy-based clearance in the liver, thereby increasing the sensitivity of cells to insulin and attenuating glucose tolerance (Fig. 6) [105]. In addition, R406 inhibits splenic tyrosine kinase pathway activation, which is related to the pathogenesis of NASH and alcoholic hepatitis. Kurniawan et al. synthesized R406-loaded PLGA NPs to improve the pharmacokinetics of R406, which ameliorated fibrosis, inflammation, and steatosis in mice, restoring liver function and reducing plasma lipid levels [107].

### Liver fibrosis

Liver fibrosis is the primary cause of mortality in patients with chronic hepatic disease [149]. The common causes of fibrosis include chronic hepatitis B or C infections, alcohol abuse, NAFLD, autoimmune liver disease, and hereditary diseases [150]. The mechanisms of liver fibrogenesis include: (i) reactive oxygen species (ROS) and other oxidative stress-related mediators induce a chronic inflammatory response to activated HSCs (aHSCs) in the liver [151, 152]; (ii) extracellular vesicles released by injured and/or apoptotic hepatocytes affect almost all cell populations, inducing/sustaining inflammation, fibrosis and angiogenesis [153]; and (iii) excess ECM induces remarkable changes in the quality and morphological distribution of ECM components, especially type I and III collagen, due to imbalanced synthesis and degradation of collagen fibres and increased expression of inhibitors of metalloproteinases (TIMPs) [154]. HSCs are activated and transformed into hepatic myofibroblasts (HMFs) after hepatic injury [153]. aHSCs play vital roles in the synthesis and secretion of excessive ECM in response to damaged hepatocytes, LSECs and KCs. Therefore, aHSCs are hallmarks of hepatic fibrosis and are known as major targets for the treatment of hepatic fibrosis [155].

Hepatic fibrosis can be attenuated either by delaying the progression of fibrosis and/or by promoting the resolution of fibrosis. The primary treatment strategy for hepatic fibrosis is withdrawal of all pathogenic microorganisms and chemicals that continuously damage the liver parenchyma [153]. Many drugs, plant and animal extracts, and monoclonal antibodies (mAbs) are being developed for treating hepatic fibrosis [12, 14, 156, 157], including IFN-γ, pirfenidone, vitamin E, colchicine, anti-CCL24 mAbs, anti-PDGF-B mAbs, polydatin, renin-angiotensin system inhibitors, silymarin, and nucleic acids [7, 158]. Nevertheless, none of these candidates are effective in stopping or reversing hepatic fibrosis in clinical trials due to insufficient drug delivery to the fibrotic liver which has changed macrostructure and microenvironment. Therefore, no agents have been approved as antifibrotic drugs [159]. To overcome the barriers to drug development, the application of nanotechnology has attracted increasing attention.

**Fig. 7:**
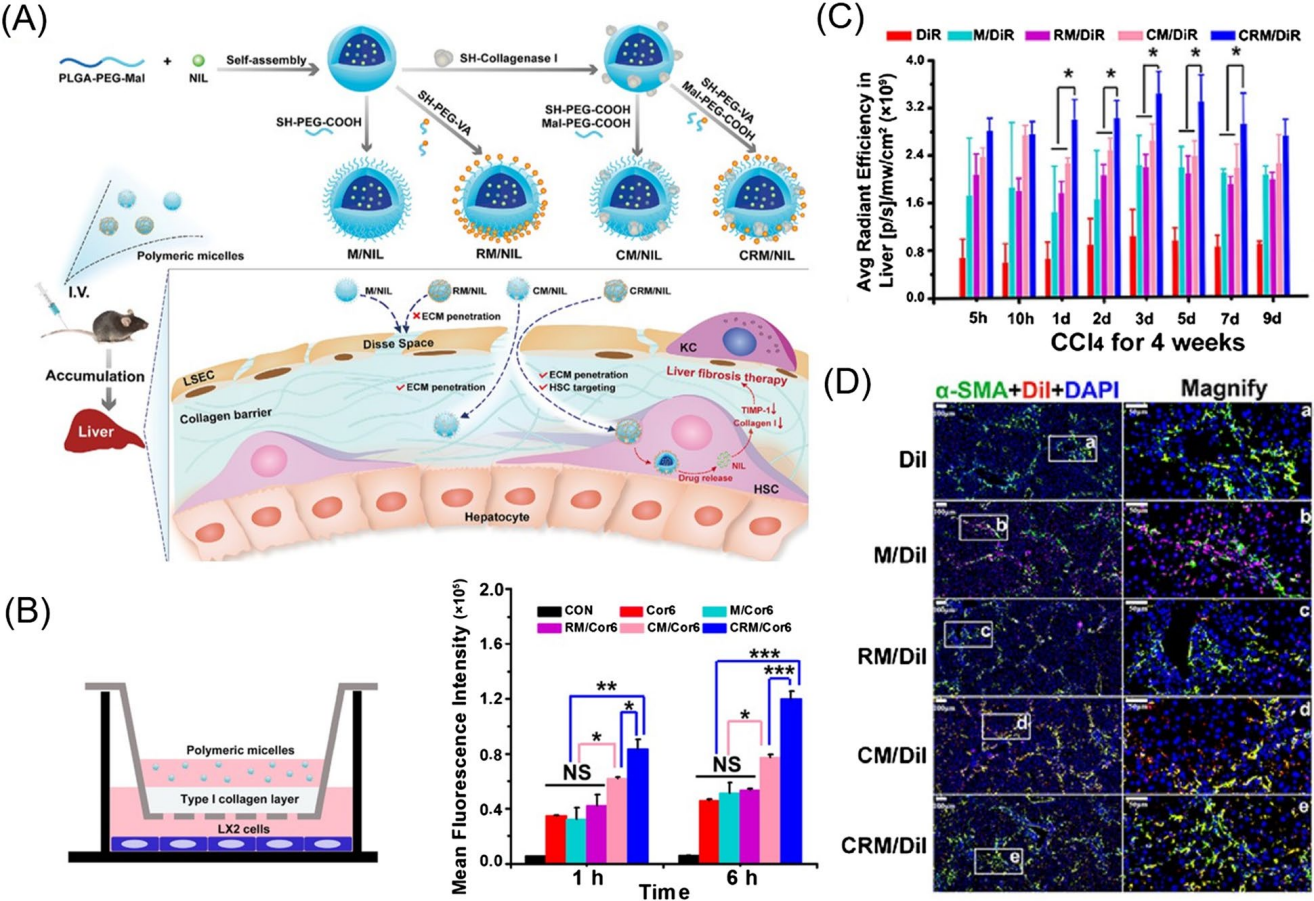
**A** Schematic illustration showing the preparation of polymeric micelles and their proposed destinations in vivo. **B** Cellular uptake of polymeric micelles through anti-collagen I barrier activity in vitro. **C** Fluorescence intensity in the liver of normal mice and mice treated with CCl4 for 4 weeks, expressed as average radiant efficiency units. **D** Colocalization of DiI and DiI-labelled polymeric micelles with activated HSCs in the liver of fibrotic mice. (Reprinted with permission from Ref. [165], Copyright 2019, Elsevier Ltd)
(iii) Nucleic acid delivery: Novel nucleic acid-based antifibrotic agents show specificity and efficacy, but their use in the treatment of fibrosis becomes a heavy burden for patients, since repetitive and long session of parenteral administration are necessary, as most nucleic acids are rapidly identified and degraded by nucleases in the bloodstream. Therefore, development of satisfactory biocompatible and biodegradable carriers to deliver nucleic acids is urgently needed. A pH-sensitive vitamin A (VA)-conjugated copolymer VA-PEG-polyethyleneimine-poly (*N*-(*N′*,*N*′-diisopropylaminoethyl)-*co*-benzylamino) aspartamide (T-PBP) was developed and assembled into superparamagnetic iron oxide-decorated cationic micelles for miRNA-29b and miRNA-122 delivery [166]. The T-PBP micelle efficiently delivered the microRNAs (miRNAs) in a manner that allowed magnetic resonance imaging (MRI). A synergistic antifibrotic effect was realized through suppressing the expression of fibrosis-related genes, including collagen type I alpha 1, ɑ-SMA, and TIMPs. This study revealed excellent antifibrotic efficacy in terms of improved liver function and alleviated liver fibrosis in vivo. Ketal cross-linked cationic nanohydrogel particles were also fabricated to deliver siRNA, which were degraded at endosomal pH and thus released siRNA. More importantly, the particles accumulated in fibrotic tissue, facilitating the knockout of sequence-specific genes related to fibrosis [167]. Zhang et al. developed a retinol-conjugated polyetherimine (RcP) NP to selectively adsorb retinol binding protein 4 (RBP) as its corona components. The RBP-incorporated NPs could deliver antisense oligonucleotide to HSCs, effectively downregulating the expression of collagen I and subsequently alleviating fibrosis [168].
(iv) Codelivery of antifibrotic agents: The synergistic therapeutic effectiveness of chemical and nucleic acid drugs has been confirmed in liver fibrosis. Qiao et al. prepared a nanomicelle with poly(lactide-*co*-glycolide)-polyspermine-PEG-vitamin A (PLGA-PSPE-PEG-VA) for the codelivery of silibinin and siCol1α1. The VA added to the surface of the NPs specifically bound to the retinol-binding protein receptor on aHSCs in the fibrotic liver, which led to more efficient reduction of collagen I production and significant alleviation of hepatic fibrosis [86]. In addition, Ji et al. reported that germacrone and miR-29b were coencapsulated into PEG-PLGA based NPs. The prepared NPs were decorated with cyclic RGD (cRGD) peptides. These NPs exhibited great ability to target the fibrotic liver in mice because of the cRGD modification, inducing high cytotoxicity in aHSCs and dramatically suppressing the production of type I collagen [88].

(i) Natural extract delivery: Natural extracts are considered sources of novel bioactive substances due to their excellent antifibrotic, antihepatotoxic and antioxidant properties [157, 160]. As hyaluronic acid (HA) can specifically bind to CD44 receptors, which are highly expressed on the surface of aHSCs, Li et al. synthesized a silibinin-loaded HA (SLB-HA) micelle to treat hepatic fibrosis. The micelles showed superior targeting to fibrotic liver and specifically bound to and killed aHSCs, leading to an excellent anti-hepatic fibrosis effect [161]. In addition, Lin et al. designed a polydatin-loaded micelle (PD-MC) based on a ROS and pH dual-sensitive block polymer PEG-poly(2-((((4-(4,4,5,5-tetramethyl-1,3,2-dioxaborolan-2-yl)benzyl)oxy) carbonyl) oxy)ethyl methacrylate co 2-(diisopropyl amino)ethyl methacrylate) (P(PBEM-*co*-DPA). The micelle shows excellent liver-targeted drug release in response to high ROS levels and acidic environments. Moreover, the PEG-p(PBEM-*co*-DPA) micelle can deplete ROS at the pathological site to exert anti-inflammatory effects. Results have shown that PD-MC can dramatically inhibit inflammatory reactions and oxidative stress, decrease hepatocyte apoptosis, and prevent the activation of macrophages and HSCs [162]. In addition to plant extracts, animal extracts have also been delivered to aHSCs as antifibrotic drugs. For instance, astaxanthin is a keto-carotenoid in the terpene class of chemical compounds, and it is abundant in marine animals, such as salmon and shrimp. Astaxanthin has shown antioxidant and anti-inflammatory activities. Hu et al. prepared biopolymer-based NPs using a stearic acid-CS conjugate (SA-CS) and sodium caseinate to load astaxanthin. These NPs dramatically enhanced LX-2 cellular bioactivity by reducing TGFβ1-induced fibrogenic gene expression levels, as well as α-SMA and COL1A1 protein levels, compared to free astaxanthin [163].
(ii) Synthetic small-molecule delivery: Tyrosine kinase inhibitors (TKIs) (e.g., sorafenib and nilotinib) exhibit powerful antifibrotic activity. Lin et al. developed a mixture of PEG-PLGA and PLGA to transport sorafenib, which increased the uptake by fibrotic liver cells and decreased α-SMA levels and collagen production. Furthermore, these NPs dramatically reduced the size of abnormal blood vessels and reduced microvascular density, restoring the normal function of the vessels in the fibrotic liver [164]. To increase the permeability of nanoformulations and accurately target aHSCs, Fan et al. developed collagenase I and retinol co-modified polymeric micelles that have nanodrill-like and HSC-targeting properties, based on PLGA-PEG-Mal, to load nilotinib for liver fibrosis therapy. This micelle efficiently degraded pericellular collagen I and exhibited increased uptake by HSCs. Moreover, these micelles showed excellent accumulation in the fibrotic liver and accurate targeting to aHSCs, showing optimal antifibrotic activity (Fig. 7) [165].

### Hepatocellular carcinoma

HCC is the most common form of hepatic cancer and accounts for ~ 90% of liver cancer cases [169]. More than 90% of HCC cases are diagnosed in patients with chronic hepatic disease, mostly as a result of hepatic inflammation [170]. In the early stages of HCC, the lesion can be removed by surgical resection, LT and nonsurgical local ablation techniques. For intermediate-stage HCC, transarterial chemoembolization (TACE) has been the most widely used treatment method for the past 20 years [171]. In addition, patients with advanced disease will first receive systemic therapies with conventional chemotherapeutics, immune checkpoint inhibitors (ICIs), TKIs or mAbs [2]. Among all the anticancer agents, sorafenib and lenvatinib remain the most effective single-drug therapies [171]. Nevertheless, combination treatments, such as the combination of ICIs with TKIs or PD1/PDL1 axis inhibitors with CTLA4 inhibitors, are promising future therapeutic strategies.

However, the insufficient distribution of drugs in tumours and the multidrug resistance of tumour cells reduce the therapeutic outcomes of anticancer agents [5]. The proper carriers can enhance the therapeutic effect and decrease side effects [45, 46, 172]. Several polymeric nanocarrier systems have shown promise in the treatment of HCC in experimental studies and will be discussed based on the classification of antitumor agents including natural extracts, chemotherapeutic drugs, and nucleic acids.

**Fig. 8:**
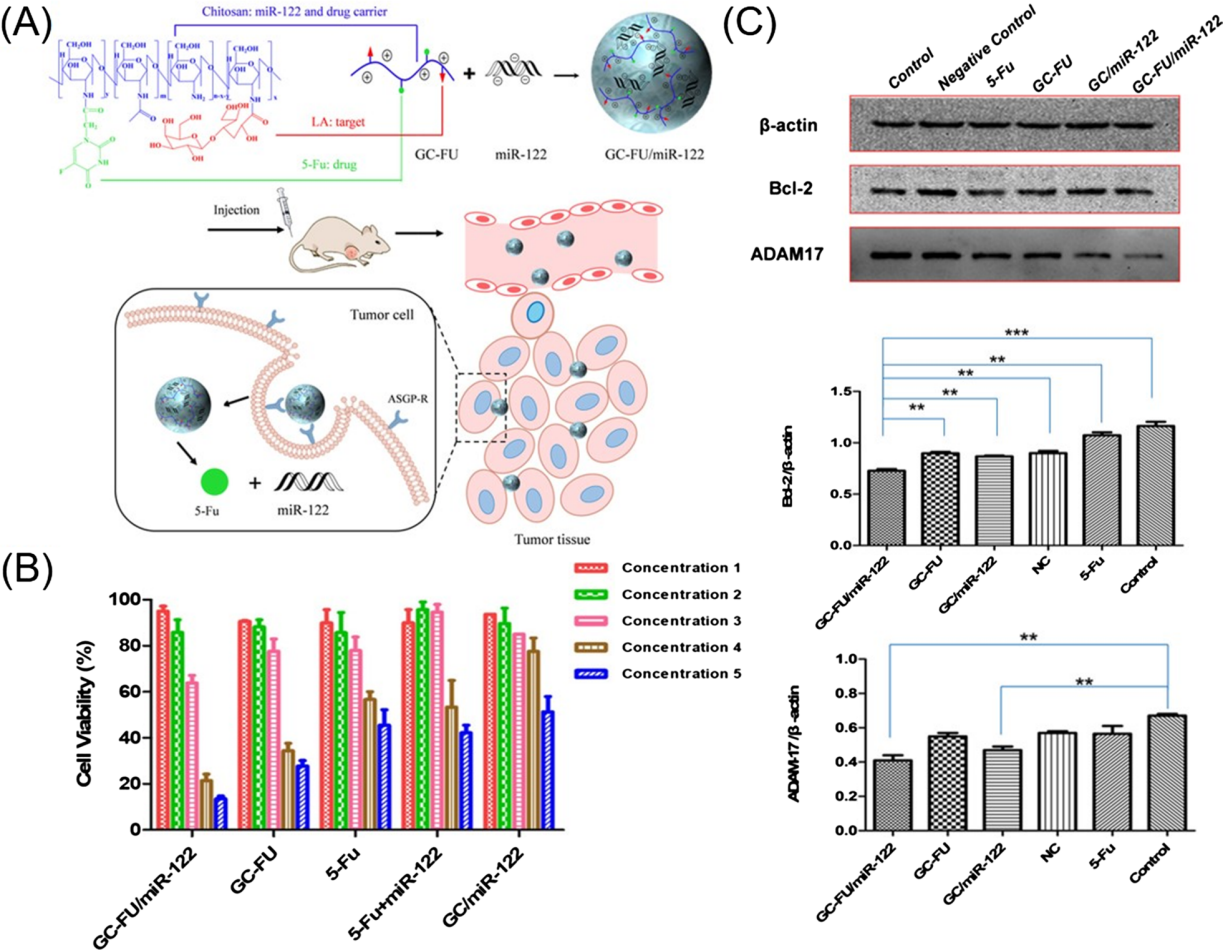
**A** Preparation of GC-FU/miR-122 and hepatoma-targeted codelivery of miR-122 and 5-Fu. **B** Viability of HepG2 cells after incubation with various treatment constructs. **C** The expression of Bcl-2 and ADAM17 in the HepG2 cells and quantitative analysis. (Reprinted with permission from Ref. [184], Copyright 2019, American Chemical Society)

(i) Natural extract delivery: Natural compounds are being investigated as anticancer drugs in view of their excellent therapeutic potentials. To maximize drug efficacy, some novel intelligent delivery nanoplatforms have recently been constructed. For example, a smart core-crosslinked camptothecin (CPT) prodrug micelle was prepared based on a phenylboronic acid-modified PEG-polyglutamic acid polymer with disulfide-bonded CPT. This micelle exhibited enhanced cellular uptake, good reduction sensitivity, and significant in vitro and in vivo antitumor efficacy [173]. Ursolic acid, a pentacyclic triterpene acid derived from many plants, has many pharmacological effects, including antioxidant, anti-inflammatory, antibacterial, anticancer, and antifungal properties. Shen et al. developed an amphiphilic self-assembled nanodrug consisting of ursolic acid, lactobionic acid and low-polyamidoamine dendrimers, which increased cytotoxicity against hepatic cancer and attenuated the migration and adhesion of SMMC7721 cells by inhibiting metastasis-related protein MMP-9 expression [56]. Polyphenols are plant-derived dietary compounds that can prevent certain chronic diseases, such as cardiovascular disease, neurodegenerative diseases, and tumours. For example, curcumin was encapsulated into the core of poly-l-lysine (PLL)-based NPs for pH-sensitive controlled release. These NPs enhanced the cellular uptake of curcumin into the human hepatoma Hep3B cell line by electrostatically absorptive endocytosis and showed prolonged blood circulation time [174].
(ii) Chemotherapeutic drug delivery: Chemotherapeutic drugs have various drawbacks, such as poor solubility and short half-life in the circulatory system. Multiple polymeric nanocarriers have recently been developed to solve these problems. Owing to their biocompatibility, biodegradability, and low immunogenicity, naturally occurring polysaccharides, such as CS-based NPs, have been used for transporting doxorubicin (DOX) [55, 175]. In one study, a CS-based nanoplatform was functionalized by dual ligands (lactobionic acid and glycyrrhetinic acid) to target HepG2 cells and improve intracellular drug uptake [55]. The use of all-trans retinoic acid (ATRA), a potent Pin1 inhibitor, in solid tumours has been limited because ATRA has a relatively short half-life in blood. Therefore, Yang et al. designed a novel formulation of ATRA based on poly-l-lactic acid for effective HCC therapy. The as-prepared formulation significantly enhanced the suppression capabilities of ATRA on HCC cell growth, increasing the half maximal inhibitory dose (IC50) by more than threefold, compared to that of free ATRA [176]. In addition, sorafenib-loaded polymeric NPs were prepared by self-assembly of TPGS-*b*-poly(caprolactone) (TPGS-*b*-PCL), Pluronic P123 and SFB, followed by conjugation with an anti-GPC3 antibody for targeted treatment of liver cancer. These antibody-conjugated NPs increased cellular uptake by HepG2 cells and enhanced cytotoxicity compared with untargeted NPs [177]. 
  Intelligently controlled release of anticancer agents based on responses to external stimuli or tumour environment stimuli has been widely leveraged. Wang et al. constructed ultrasound-responsive drug delivery NPs based on poly(lactobion-amidoethyl methacrylate) to achieve on-demand ultrasound-induced DOX release [178]. Yan et al. developed a smart acid-responsive micelle based on glycyrrhetinic acid-modified CS-polyethyleneimine-4-hydrazinobenzoic acid-DOX for the targeted delivery and pH-responsive release of DOX, which resulted in efficient targeted killing of HepG2 cells [179]. Similarly, redox-responsive theranostic NPs based on poly-(*N*-ε-carbobenzyloxy-l-lysine) (PZLL)-grafted HA copolymers were designed for the targeted delivery of superparamagnetic iron oxide (SPIO) and DOX for use in HCC diagnostics and therapy; this method made it easy to obtain real-time information on the biodistribution of DOX to quantitatively measure intracellular drug uptake and evaluate the treatment effect on cancer [68].
(iii) Nucleic acid delivery: MiRNA-122 (miR-122) can inhibit hepatocarcinogenesis and progression, is prone to degradation in the blood and cannot effectively accumulate at a tumour site. Therefore, Guo et al. developed an ultrasound-triggered phase-transitioning and size-changing cationic nanodroplet, perfluoropentane/C9F17-PAsp(DET)/miR-122/poly(glutamic acid)-g-MeO-PEG ternary nanodroplets, to deliver miR-122. The nanodroplets combined with ultrasound radiation significantly inhibited the growth, migration, and invasion of HCC cells [180]. In addition, short GC-rich DNA (GCD) interfered with the polymerization of microtubules and induced cell apoptosis and cell cycle arrest, resulting in effective tumour cell killing. Yang and coworkers synthesized phenylboronic acid-modified polyamidoamine and used it to deliver GCD, leading to effective suppression of cell migration and invasion [181]. Similarly, a novel copolymer composed of low-molecular-weight polyethylenimine (PEI) cross-linked with myoinositol and conjugated with a galactose-grafted PEG chain was synthesized to deliver a plasmid encoding the IL15 gene. These polyplexes showed improved transfection efficiency, effectively suppressing tumour growth and prolonging the survival time of tumour-bearing mice after intraperitoneal injection, and are, therefore, a promising gene delivery system for immunogene therapy in HCC [182].
(iv) Codelivery of antitumor agents: Sorafenib (SFB) has been the only available standard of care for advanced HCC for a decade. However, it is prone to induce resistance in liver cancer cells, as mentioned above. Codelivery of a small-molecule inhibitor of the PI3K/mTOR pathway (BEZ235) and SFB increased SFB therapeutic effectiveness against HCC and, notably, enhanced the treatment effectiveness of SFB-resistant HCC [183]. The strategy for efficient codelivery of nucleic acids and drugs is profoundly and negatively affected by premature drug leakage in circulation and serious off-target-associated side effects. Ning et al. reported the codelivery of hepatic-specific miR-122 and the antitumor agent 5-fluorouracil (5-Fu) by exploiting a macromolecular prodrug pathway. The complexes showed increased stability, efficiently inhibited the growth of tumour cells, further induced the apoptosis of HCC cells, and downregulated the expression of ADAM17 and Bcl-2 (Fig. 8) [184]. P-glycoprotein (P-gp) promotes drug efflux, while decreasing pro-survival Bcl-2 expression is an important goal in the treatment of liver cancer. To this end, Cheng et al. employed an amphiphilic poly[(R)-3-hydroxybutyrate] (PHB)-b-poly(2-(dimethylamino)ethyl methacrylate) (PDMAEMA) cationic polyester to deliver paclitaxel (PTX) and the Bcl-2 convertor Nur77/ΔDBD gene to effectively suppress drug-resistant HepG2/STC2 and SMCC7721/STC2 cell proliferation, partially erode P-gp-induced PTX efflux and activate the apoptotic function of the pro-survival protein Bcl-2 [185].

### Host-versus-graft disease

LT is the only effective treatment method for end-stage hepatic diseases, such as acute hepatic failure, a life-threatening systemic complication of hepatic disease, a hepatic-based metabolic disorder, or cirrhosis with complications including HCC, variceal haemorrhage induced by portal hypertension, ascites, hepatorenal syndrome and encephalopathy [186, 187]. However, HVGD, which is the most common type of immune rejection after LT, can lead to abnormal liver function and even graft dysfunction [188, 189]. Therefore, the lifelong use of immunosuppressants is necessary for patients to inhibit transplant rejection. The commonly used immunosuppressants include calcineurin inhibitors (cyclosporine (CsA) and tacrolimus (Tac)), glucocorticoids, cytostatics (azathioprine (AZA) and mycophenolate mofetil (MMF)), and mTOR inhibitors (sirolimus (SRL) and everolimus (EVR)) [187]. Nevertheless, immunosuppressants exhibit certain problems, such as a narrow therapeutic window, poor aqueous solubility, low bioavailability, and significant individual differences. In addition, immunosuppressants can cause severe side effects, such as infection, nephrotoxicity, hepatotoxicity, de novo tumours, pneumonitis, and bone marrow suppression [187, 190]. Nanocarriers can effectively improve the physicochemical properties of immunosuppressants, mediate the targeted delivery of immunosuppressive agents to liver tissues, and reduce the side effects of the agents. Recent development of polymer carriers for the delivery of immunosuppressants is discussed in this section.

(i) Immunosuppressant delivery: Tac blocks the production of interleukin-2 (IL-2), inhibiting T-cell activation and proliferation at an early stage. However, the poor solubility and instability of Tac hinders its clinical use. Therefore, Tac-loaded micelles were developed with various biodegradable polymers, such as poly(ɛ-caprolactone)-PEG-poly(ɛ-caprolactone), PEG-poly(epsilon-caprolactone), PEG-poly(d,l-lactide), poly(methyl vinyl ether-*co*-maleic anhydride)-graft-hydroxypropyl-β-cyclodextrin, and an ethyl cellulose (EC) polymer, to improve the solubility, bioavailability, and blood circulation of Tac [191–196]. MMF is an antiproliferative immunosuppressant drug that can be converted to the active ingredient mycophenolic acid (MPA) in the liver and intestinal wall. A high MPA dose can lead to adverse effects such as diarrhoea, nausea, and vomiting. Mohammed et al. developed an oral formulation of MMF for once-daily dosing using CS-coated PLA or PLGA NPs, and they showed higher envelopment and drug release rates [197]. HVGD after LT is an immune disease mediated by T cells. Therefore, targeted delivery of Tac to T cells leads to precise intervention. A targeted delivery platform based on PLGA NPs decorated with CS and the CD8AP17s aptamer (Apt) was designed to efficiently transport Tac into MOLT-4 cells and reduce off-target cell toxicity [130].
(ii) Nucleic acid delivery: Toll-like receptors (TLRs) play crucial roles in the induction of allograft rejection. Myeloid differentiation factor 88 (MyD88) is a fundamental adaptor in TLR signalling, and inhibiting the expression of MyD88 can prolong the survival of allografts. Therefore, Hu et al. developed a histidine-grafted poly(β-amino ester) (HGPAE) nanocarrier to deliver a plasmid containing MyD88-targeting short hairpin RNA (shRNA). The pMyD88/HGPAE complexes significantly inhibited the expression of MyD88 in rat hepatic tissue, prolonged allograft survival and significantly reduced the serum levels of IL-2 and IFN-γ in the recipients [127].

## The fate of NPs in the liver

**Fig. 9:**
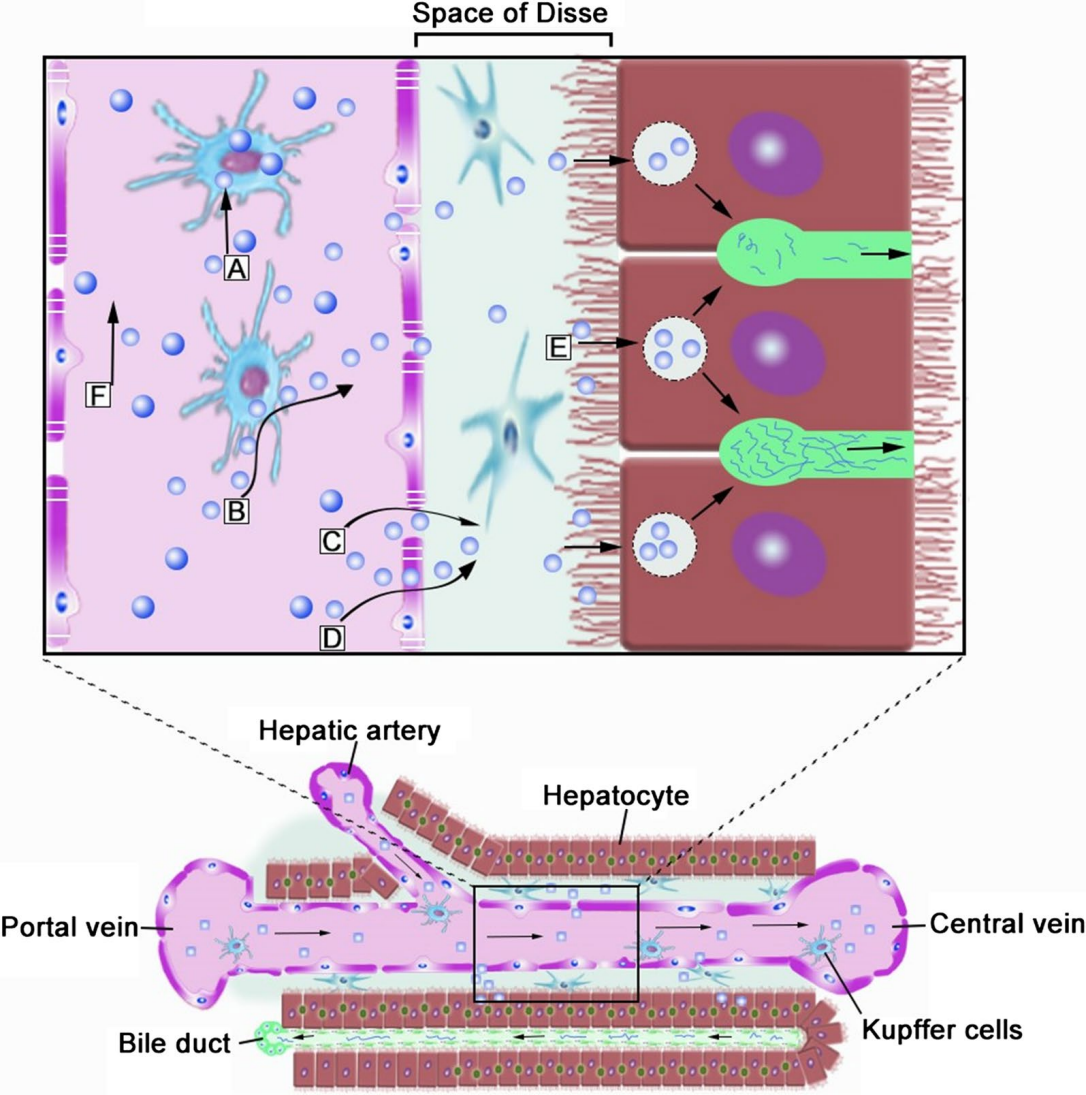
Proposed elimination mechanism of biodegradable nanoparticle (NP) in the liver. Intravenously injected NPs enter the liver and into hepatic sinusoids. **A** Kupfer cells (KCs) take up the majority of circulating NPs on the basis of NP size. **B** NPs can escape from KCs. **C**, **D** NPs that are smaller than the diameter of liver sinusoidal fenestrations (up to 100–200 nm) can enter the space of Disse through LSECs or fenestrations. **E** NPs then collect in the space of Disse, where hepatocytes slowly internalize and process them for transport into the bile canaliculus. **F** Larger NPs may not be able to enter fenestrations or access by LSECs and thus continue to circulate throughout the body. (Reprinted with permission from Ref. [200], Copyright 2019, American Chemical Society)

As NP-based technologies continue to be developed for the diagnosis and therapy of liver diseases [8, 11, 12], the need to understand the intrahepatic distribution and potential clearance mechanisms of NPs is ongoing. The liver, as the largest organ RES in the body, can sequester 30–99% of NPs delivered through the blood circulation system [38]. The fate of these NPs, as reported, includes (i) cellular uptake by KCs. KCs form the first line of liver defence and contribute to substance clearance through the action of scavenger receptors [198]. Therefore, KCs are more likely than others to phagocytose NPs [199, 200]. Once being internalized, NPs may be processed by autophagy and endolysosomal pathways. Autophagy and endolysosomal pathways effectively isolate particles from the surrounding environment, degrade the nanomaterials, reduce the associated toxicity and aid in decreasing cellular stress [201]; (ii) Hepatobiliary elimination. Circulating NPs that are not internalized by KCs or that escape from KCs but are smaller than the diameter of liver sinusoidal fenestrations (up to 100–200 nm), can enter into the space of Disse through the fenestrae or LSECs. Subsequently, they are phagocytosed by hepatocytes, degraded by various enzyme systems, and excreted into the biliary tract [38, 202, 203] (Fig. 9).

However, the distribution and metabolism of NPs change due to the changes of liver anatomy and function in some liver diseases. For example, owing to the capillarization of sinusoids and deposition of ECM in liver fibrosis and cirrhosis, NPs cannot readily enter the space of Disse and thus cannot be taken up by hepatocytes or HSCs to exert therapeutic functions [7, 153]. In contrast, NPs can accumulate in liver tumour by EPR effect as mentioned above and then can be internalized by tumour cells. Therefore, to develop effective nanomedicines to treat certain liver diseases, the relationships between NPs and the liver from an organ-to-cell perspective need a deeper investigation.

## Conclusions and prospects

Liver diseases pose a serious threat to human health. Nevertheless, conventional medications cannot be delivered to the location of diseases at a sufficient concentration and may cause significant adverse effects. In recent years, various polymer-based nanomedicines have been designed and developed to carry small molecules, peptides and nucleic acids for the diagnosis and treatment of hepatic diseases, resulting in improved therapeutic outcomes and alleviated systemic toxicity. However, the clinical application of polymer nanomedicines is still limited. Several challenges hinder the clinical translation of polymeric nanomedicines and need to be addressed.

First, many potential therapies have been effective in animal models, but their therapeutic efficacy in humans has been less than satisfactory. Several issues may contribute to these outcomes: (i) The types and number of receptors on liver cells are not entirely known, and ligands applied in animal experiments may not specifically recognize human receptors; (ii) NPs, as exogenous substances, exert immunogenetic effects after entering the systemic circulation and are readily internalized by immune cells; hence, it is difficult to deliver drugs to target cells where they can exert their pharmacological effects; and (iii) the animal models cannot mimic the clinical situation. In humans, fibrosis, cirrhosis, and even HCC develop over decades, and symptoms primarily develop in the mid and late stages of the disease. However, these processes develop in weeks or months in rodents, and therefore, the pathology has less time to ‘mature’. More importantly, many treatment experiments have been conducted at earlier stages of the disease, and it seems that diseases in rodents are cured more easily than human liver diseases. Therefore, improved animal models need to be established to better investigate the therapeutic effects of nanomedicines.

Second, polymeric micelles are promising platforms to improve the bioavailability of free drugs. However, after being administered into the bloodstream, micelles are affected by a variety of factors, including temperature, pH, ionic strength, and biological molecules, leading to the instability of the micelle structure [204]. Therefore, it is necessary to improve the stability of micelles to reduce premature agent release in the bloodstream. Studies have shown that dynamic covalent or noncovalent crosslinking can effectively reinforce the structure and improve the stability of micelles [205, 206]; therefore, crosslinked polymer micelles are expected to be promising delivery systems. Improving the controllability of the molecular structure and optimizing the preparation methods of polymer micelles are hotspots for future research. In summary, the development of polymeric carriers with novel materials and technologies is critical, and investigations into the best mechanism for fabrication of micelles as efficient drug delivery systems are important.

Third, a major challenge involves the failure of many NPs to reach targeted liver cells. The liver is the largest organ RES and can sequester 30–99% of administered NPs in the blood circulatory system. However, most NPs, as xenobiotics, are located in KCs, even when targeted ligands are added to the NPs. In addition, some NPs, which escape from KCs, enter the space of Disse and are phagocytized by hepatocytes or HSCs. However, we do not know whether these NPs are deformed or fragmented as they pass through the fenestrations. Therefore, the mechanism by which NPs extravasate into the space of Disse needs further investigation. To reduce the uptake of NPs by the RES and prolong the circulation of the NPs in the bloodstream, PEG chains have been widely used to modify the nanomedicine surface [207, 208]. In addition, although most ligand-modified NPs can be internalized by major target cells in vitro and can accumulate in the liver in vivo, no direct evidence has shown how these NPs can be phagocytized by target cells in vivo and perform effectively. Therefore, interest in using polymers to transport contrast agents for MRI and computed tomography (CT) is increasing, and nanocarriers can co-deliver SPIO and therapeutic agents, enabling dynamic monitoring of the location and effects of nanodrugs [166, 209]. This direction aligns with the current trend towards personalized medicine, where the best treatment schedule and dose vary by individual patient. In addition, the application of radiolabelled constructs and immunohistochemical analyses of tissues from multiple organs can be used to track the trajectory of NPs. Therefore, the application of biodegradable multifunctional composite nanocarriers, with the addition of rigid security and efficacy testing, may provide accurate and effective treatment for hepatic diseases and will be a significant direction for future nanomedicine studies.

Fourth, liver cell-targeted delivery and stimulus-responsive release have become important research aspects in the field of drug delivery. As mentioned above, each disease is characterized by target cells, and nearly all resident liver cells can be reached by applying different carriers. However, the liver, as the largest organ RES, can take up most NPs, and therefore, it is essential to verify that targeted NPs confer better healing than untargeted NPs in vivo. In addition, developing environment-responsive nanomedicines, which can intelligently respond to endogenous (redox, pH, or enzyme) or exogenous stimuli (temperature, ultraviolet light, near infrared light, ultrasound, or magnetic fields) and release payload at targeted sites, will increase therapeutic efficacy for diseased/damaged cells and reduce toxicity to normal cells [210]. Active targeting and environment responsiveness can realize the spatio-temporally controlled drug release, which will be the hotspots in future nanomedicine research. To advance the development of nanomedicine, we need to pay greater attention to the pathophysiology of liver disease and develop reasonable drug carriers capable of differentiating between normal cells and diseased/damaged cells at both the cellular and gene levels. To gain the needed understanding, the joint efforts of chemists, pathologists, physiologists, and other professionals are required.

Finally, rigorous biosafety assessments need to be established to evaluate the immunogenicity of polymer NPs, the safety of NP degradation products, NP effects on hepatocyte functions, and the pharmacokinetics of the medicinal systems. The long-term effects of NPs need to be carefully and systemically evaluated because patients with liver diseases present with low immunity and lack of self-repair ability. Therefore, the safety risks involved in the application of NPs for the treatment of hepatic disease need to be given great attention. Furthermore, liver diseases have a long course, and sensitive methods to continuously monitor changes in liver function during disease treatment are lacking; therefore, sensitive serum markers that can be used to assess the progress of disease and measure the effectiveness of drugs need to be extensively explored.

With the advances in chemistry, biology, nanotechnology and medicine, deeper insights into the pathophysiology of liver diseases, and the emergence of new analysis methods and design concepts, we believe that novel polymeric nanomedicines will be developed to address the aforementioned challenges. As a result, the therapeutic outcomes of polymeric nanomedicines are expected to be improved, thus promoting their clinical translation in the treatment of hepatic diseases.

## Data Availability

Not applicable, please refer to the original references.

## Abbreviations

HCC: Hepatocellular carcinoma
HBV: Hepatitis B virus
LT: Liver transplantation
NPs: Nanoparticles
NASH: Nonalcoholic steatohepatitis
HVGD: Host-versus-graft disease
PLGA: Poly(lactic-*co*-glycolic acid)
NPCs: Nonparenchymal cell
LSECs: Liver sinusoidal endothelial cells
HSCs: Hepatic stellate cells
KCs: Kupffer cells
NAFLD: Nonalcoholic fatty liver disease
RES: Reticuloendothelial system
ECM: Extracellular matrix
aHSCs: Activated HSCs
PEG: Polyethylene glycol
EPR: Enhanced permeability and retention
HBsAg: Hepatitis B surface antigen
CS: Chitosan
PCL: Poly-ε-caprolactone
BMDCs: Bone marrow-derived dendritic cells
Ngs: Nanogels
γ-PGA: Poly-γ-glutamic acid
pDNA: Plasmid DNA
CPPs: Cell-penetrating peptides
PNAs: Peptide nucleic acids
CSSO: CS oligosaccharide-SS-octadecylamine
LPH: Lipid polymer hybrid
NAFL: Nonalcoholic fatty liver
Res: Resveratrol
Gal: Galactose
DNL: De novo lipogenesis
SREBP: Sterol regulatory element-binding protein
RAPA: Rapamycin
FNB: Fenofibrate
NFD: Nifedipine
HFD: High-fat diet
Lac-PDMAEMA: Lactosylated poly(*N*,*N*-dimethylaminoethyl methacrylate)
PPARγ: Peroxisome proliferator-activated receptorγ
ROS: Reactive oxygen species
TIMPs: Tissue inhibitors of metalloproteinases
HMFs: Hepatic myofibroblasts
mAbs: Monoclonal antibodies
HA: Hyaluronic acid
SLB: Silibinin
P(PBEM-*co*-DPA): Poly(2-((((4-(4,4,5,5-tetramethyl-1,3,2-dioxaborolan-2-yl)benzyl)oxy) carbonyl) oxy)ethyl methacrylate *co* 2-(diisopropyl amino)ethyl methacrylate)
SA: Stearic acid
TKIs: Tyrosine kinase inhibitors
VA: Vitamin A
miRNAs: MicroRNAs
MRI: Magnetic resonance imaging
RcP: Retinol-conjugated polyetherimine
RBP: Retinol binding protein 4
PSPE: Polyspermine
cRGD: Cyclic RGD
TACE: Transarterial chemoembolization
ICIs: Immune checkpoint inhibitors
CTLA4: Cytotoxic T-lymphocyte antigen 4
CPT: Camptothecin
MMP-9: Matrix metalloproteinase-9
PLL: Poly-l-lysine
DOX: Doxorubicin
ATRA: All-trans retinoic acid
PCL: Poly(caprolactone)
PZLL: Poly-(*N*-ε-carbobenzyloxy-l-lysine)
SPIO: Superparamagnetic iron oxide
PEI: Polyethylenimine
SFB: Sorafenib
5-Fu: 5-fluorouracil
P-gp: P-glycoprotein
PTX: Paclitaxel
PDMAEMA: Poly(2-(dimethylamino)ethyl methacrylate)
CsA: Cyclosporine
Tac: Tacrolimus
AZA: Azathioprine
MMF: Mycophenolate mofetil
SRL: Sirolimus
EVR: Everolimus
MPA: Mycophenolic acid
Apt: Aptamer
TLRs: Toll-like receptors
MyD88: Myeloid differentiation factor 88
HGPAE: Histidine-grafted poly(β-amino ester)
shRNA: Short hairpin RNA
CT: Computed tomography
